# AGING EDUCATION IN THE UNITED STATES: WHERE WE ARE AND HOW DID WE GET HERE?

**DOI:** 10.1093/geroni/igad104.1128

**Published:** 2023-12-21

**Authors:** Laura Donorfio

**Affiliations:** University of Connecticut, Waterbury, Connecticut, United States

## Abstract

Aging education at the precollege level is relatively new in the United States, making its debut in the literature in the 1960s (Myers, 1977). The first White House Conference on Aging (WHCoA) in 1961, centered on the need for aging education and recommended it be integrated into curricula in public schools, higher education institutions, and libraries (McGuire, 2017). At the 1971 WHCoA it was noted that such educational efforts were indeed rare and again recommended that aging and life cycle education be mandatory at all levels of education, programs be developed to train teachers about aging, and older citizens be used as school volunteers (McGuire, 2017). Since then, aging curricula and intergenerational programming have made their way slowly and insufficiently into education at both the pk-12 and college levels. Interestingly, more recently, it seems more for-profit organizations are investing in aging education and training versus educational institutions to educate their workforce to better meet the needs of their older consumer base to increase their profit margins. Beyond profit margins, what is the heuristic value of aging education and why is it increasingly important and needed not only in the United States, but worldwide? This paper will kick off the symposium and set the stage with a retrospective account of what factors have propelled aging education in the United States and what we have learned to move aging education forward at both the pk-12 and higher education levels.

